# Models in biology: lessons from modeling regulation of the eukaryotic cell cycle

**DOI:** 10.1186/s12915-015-0158-9

**Published:** 2015-07-01

**Authors:** John J. Tyson, Béla Novák

**Affiliations:** Department of Biological Sciences, Virginia Polytechnic Institute & State University, Blacksburg, VA 24061 USA; Oxford Centre for Integrative Systems Biology, Department of Biochemistry, University of Oxford, Oxford, OX1 3QU UK

**Keywords:** Mathematical models, Mitosis-promoting factor, Cell cycle checkpoints, Regulated kinases, Regulated phosphatases, Regulated proteolysis

## Abstract

In this essay we illustrate some general principles of mathematical modeling in biology by our experiences in studying the molecular regulatory network underlying eukaryotic cell division. We discuss how and why the models moved from simple, parsimonious cartoons to more complex, detailed mechanisms with many kinetic parameters. We describe how the mature models made surprising and informative predictions about the control system that were later confirmed experimentally. Along the way, we comment on the ‘parameter estimation problem’ and conclude with an appeal for a greater role for mathematical models in molecular cell biology.

## Accurate descriptions of our pathetic thinking about nature

Jeremy Gunawardena [1] opened this series of review articles (on ‘models in biology’) with a quote from Nobel Laureate James Black, who described mathematical models as "accurate descriptions of our pathetic thinking about nature". Black, who made skilful use of mathematical models on the road to discovering antagonists of β-adrenergic receptors, was certainly not equating mathematical models with ‘pathetic thinking’ (as some people believe) but rather suggesting that mathematical models could be useful in turning our provisional ideas about molecular biology into real knowledge about living cells. Black saw three essential roles for models in biology: to expose assumptions, to define expectations, and to devise new tests. Our purpose in this review is to illustrate these principles by our experiences in modeling the control of mitosis in eukaryotic cells. We structure our review around subheadings derived from some cogent observations by Gunawardena (slightly paraphrased) about the art of building mathematical models in biology [1].

## Focus on the biology by asking a specific question

We focus in this review on the molecular mechanisms controlling entry into and exit from mitosis (M phase) in the natural cell cycles of frog embryos and fission yeast cells and in the artificial mitotic cycles exhibited by frog egg extracts. Because our focus is on certain principles of model building rather than a comprehensive description of cell cycle controls, we will not discuss the mechanisms controlling commitment of cells to DNA replication (S phase). Following an ancient tradition of cell biologists, we refer to G1, S and G2 phases of the cell cycle simply as ‘interphase’.

To describe the role of mathematical modeling in this endeavor, we start by summarizing the relevant knowledge — in 1990 — of the physiology and biochemistry of M-phase controls in frog eggs and fission yeast cells. Our description is based loosely on review articles of the time by Murray and Kirschner [2] and by Nurse [3].

Immature frog oocytes, which are arrested in G2 phase of meiosis I, can be induced by progesterone to proceed through meiosis I and arrest again in metaphase of meiosis II. These mature oocytes are ready for fertilization, which induces entry into a sequence of 12 rapid, synchronous, mitotic cycles culminating in the midblastula transition. Masui and Markert [4] demonstrated that the progesterone signal can be bypassed by injecting immature oocytes with cytoplasm from mature oocytes, which (they opined) contained a ‘maturation promoting factor’ (MPF) that triggered the activation of a pool of inactive MPF in the immature oocyte. Later experiments by Kirschner and colleagues [5] showed that ‘MPF activity’ is elevated whenever frog eggs are in M phase (meiosis in oocytes or mitosis in fertilized eggs), and the acronym was reinterpreted as ‘M-phase promoting factor’.

**Fig. 1:**
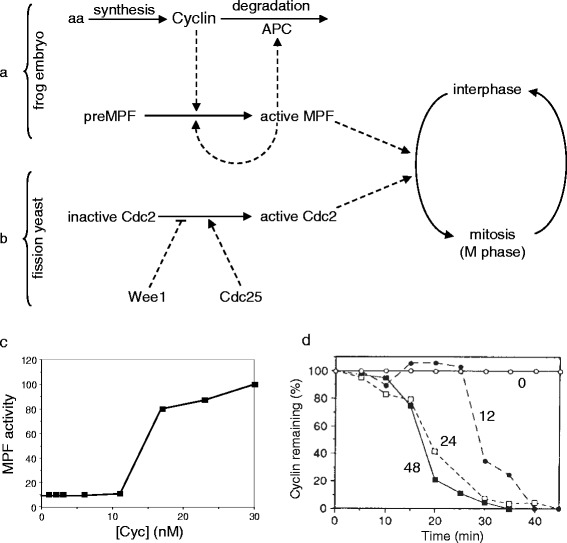
Molecular aspects of mitotic control in eukaryotic cells. **a** Biochemists’ view of the regulation of M-phase promoting factor (MPF) in early frog embryos. Derived from the discussion in [2]. Cyclin is synthesized from amino acids (aa) and degraded by a ubiquitin-dependent pathway, catalyzed by the anaphase promoting complex (APC). Cyclin promotes the activation of MPF, which enhances its own production in an autocatalytic fashion. MPF promotes the interphase-to-metaphase transition and activates the APC. **b** Geneticists’ view of Cdc2 activation in fission yeast cells. Derived from the discussion in [3]. Active Cdc2 promotes the interphase-to-metaphase transition. The activation of Cdc2 is inhibited by Wee1 and promoted by Cdc25. **c** Solomon’s signal-response curve. Adapted from figure 4B of [12]. A fixed amount of non-degradable cyclin B was added to an interphase-arrested frog egg extract (all protein synthesis is inhibited by cycloheximide). The resultant activity of MPF (the Cdc2:cyclin B heterodimer) was measured as the rate of phosphorylation of histone H1. Notice that there is a distinct cyclin threshold (between 12 and 15 nM) for activation of MPF. **d** Félix’s experiment. Adapted from figure 1A of [13]; used by permission. An interphase-arrested frog egg extract is doped with a small amount of [^35^S]cyclin B and then supplemented with increasing amounts of exogenously synthesized MPF. The resultant activity of APC is evident from the rate of degradation of the [^35^S]cyclin added to the extract. For MPF concentrations between 0 and 6 U/μl (*open circles*), the rate of cyclin degradation is minimal, and the ‘cyclin remaining’ at each time point is set to 100 %. For larger MPF concentrations, ‘cyclin remaining’ is relative to these control points. For [MPF] = 12 U/μl (*closed circles*), the [^35^S]cyclin is rapidly degraded after a time lag of ~25 minutes. For larger concentrations of MPF, 24 U/μl (*open squares*) and 48 U/μl (*closed squares*), cyclin is degraded at the same high rate, but the time lag decreases to ~10 minutes

Using frog egg extracts, which recapitulate in vitro many features of MPF activation and inactivation, Murray and Kirschner, among others, showed biochemically that the rate-limiting step for MPF activation in extracts is the synthesis of a single protein, cyclin B, and that MPF inactivation, as the extract returns to interphase, is associated with cyclin B proteolysis by a ubiquitin-dependent pathway [6, 7]. (The anaphase promoting complex, APC, is the ubiquitin-conjugating enzyme that promotes cyclin degradation as cells exit mitosis.) In addition, MPF accumulates in cells during interphase in an inactive form (preMPF); then, as cells enter mitosis, preMPF is converted to active MPF in an autocatalytic (self-amplifying) process. These discoveries led to a picture of M-phase control in frog embryos similar to the diagram in Fig. 1a.

Meanwhile, Nurse and colleagues, studying cell growth and division in fission yeast by genetic tools, had discovered three genes involved in regulating entry into mitosis: *cdc2*, *cdc25* and *wee1*. The genetic evidence suggested that the *cdc2* gene product, the protein Cdc2, can be either ‘inactive’ (in interphase) or ‘active’ (in mitosis). Cdc25, the product of the *cdc25* gene, appeared to promote the activation of Cdc2, and Wee1 to inhibit the activation of Cdc2. Significantly, Nurse found that the length of rod-shaped fission yeast cells when they enter mitosis is sensitively dependent on the dosage of the *wee1* and *cdc25* genes. Deletion of *wee1* makes cells smaller, whereas overexpression of *wee1* makes cells larger. Deletion and overexpression of *cdc25* have the opposite effects. These facts are consistent with the notion that Wee1 is an inhibitor of entry into mitosis and Cdc25 is an activator. Upon cloning these genes, Nurse and coworkers found that Cdc2 is a serine/threonine protein kinase and Wee1 is a tyrosine/threonine protein kinase. The amino acid sequence of Cdc25 was unlike any other proteins known at that time. With characteristic caution, Nurse [3] diagrammed the genetic evidence as in Fig. 1b, although he speculated that Wee1 inhibited Cdc2 by phosphorylating it on neighboring threonine and tyrosine residues, and Cdc25 activated Cdc2 by promoting the removal of these phosphate groups.

The activity called MPF proved particularly difficult to purify biochemically, but this feat was eventually achieved by Lohka et al*.* [8], who showed that MPF activity was associated with co-purification of Cdc2 and cyclin B [9–11]. It was thought most likely that cyclin binds to Cdc2 to form a kinase-active heterodimer (a process called ‘stoichiometric’ activation), but the possibility that cyclin played a catalytic role in the activation of Cdc2 could not be ruled out.

Two other experiments are especially relevant to our story. Mark Solomon, working in Kirschner’s lab, was studying the activation of Cdc2 by cyclin B in frog egg extracts that lacked both cyclin synthesis and cyclin degradation [12]. Solomon supplemented these extracts with fixed concentrations of non-degradable cyclin B and measured the resultant activity of Cdc2 kinase (by its phosphorylation of a representative substrate, histone H1) as a function of cyclin B level. We shall call this a ‘signal-response’ curve: cyclin B level being the signal, and Cdc2-kinase activity being the response. Solomon found a distinct ‘cyclin threshold’ for Cdc2 activation (Fig. 1c). For cyclin concentrations less than the threshold, he observed only background levels of histone H1 phosphorylation, but as soon as the cyclin level was greater than the threshold, the extract abruptly exhibited a high rate of histone phosphorylation, which increased further with increasing cyclin concentration.

In a similar fashion, Marie-Anne Félix was studying Cdc2-induced degradation of cyclin in frog egg extracts in Karsenti’s lab [13]. To an interphase-arrested extract (no cyclin synthesis), she added a small amount of [^35^S]-labeled cyclin B plus a measured amount of exogenously synthesized Cdc2:cyclin B heterodimers (i.e., MPF, measured in units of kinase activity per microliter of extract). She found that for MPF activity less than 6 U/μl, the [^35^S]-labeled cyclin B was only slowly degraded, but for higher concentrations of MPF (≥12 U/μl), the radioactively labeled cyclin B was rapidly degraded, after a time delay of 15 minutes or longer (Fig. 1d).

In the next section, we describe three simple mathematical models of these interactions between Cdc2 and cyclin B. The strengths and weaknesses of these models, in light of the experimental results of the time, set the stage for later, more successful models.

## We never bother ourselves with all the details

**Fig. 2:**
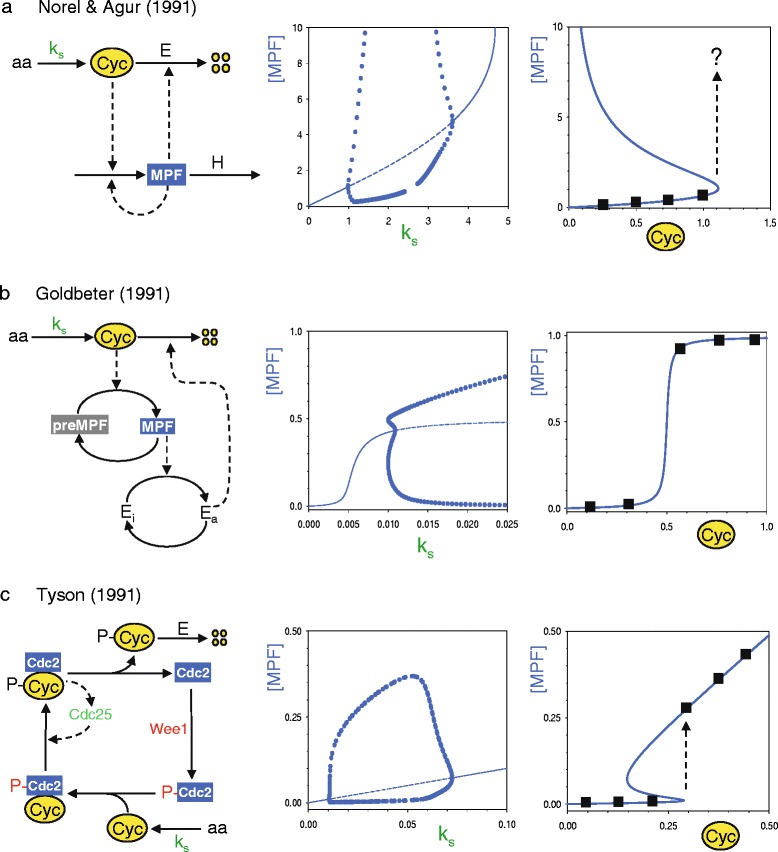
Three early models of the mitotic control system proposed by Norel and Agur [14] (**a**), Goldbeter [15] (**b**), and Tyson [16] (**c**). *Left panels*: Molecular mechanisms. *Solid arrows* represent chemical reactions; *dashed arrows* represent catalytic activities. *E* and *H* are enzymes that catalyze particular reactions; *k*_s_ is the rate constant for cyclin synthesis; −*P* indicates a phosphorylated protein. *Middle panels*: Oscillatory ranges. As a function of increasing rate of synthesis of cyclin, we plot MPF activity of each model for two types of solutions. The *solid* (*dashed*) *lines* correspond to stable (unstable) steady state solutions of the model’s differential equations. The *blue circles* correspond to the maximum (upper) and minimum (lower) activity of MPF during an oscillatory solution for a particular value of *k*_s_. Notice that oscillatory solutions are observed only over a range of values of *k*_s_. *Right panels*: Signal-response curves. For a fixed concentration of cyclin, we plot the steady state activity of MPF as predicted by each model. *Black squares* represent Solomon’s observations (adapted from Fig. 1c). In (a) and (c) the *dashed up-arrow* indicates the cyclin level where the control system would make an abrupt jump to a state of high MPF activity. In the Norel-Agur model, the MPF activity increases without bound (indicated by the *question mark*)

In 1991 three competing models of the cyclin-MPF network were published by Norel and Agur [14], Goldbeter [15], and Tyson [16]. Basing their models loosely on the experimentalists’ informal diagrams sketched in Fig. 1a, b, all three modelers focused on certain features of the network that they thought to be most important (Fig. 2a–c, left), while neglecting other biochemical details known (or suspected) at the time. For instance, Norel and Agur assumed that cyclin drives the production of MPF in a catalytic manner and that MPF activates its own production (‘autocatalysis’). Norel and Agur also assumed that MPF activates cyclin degradation (by activating the APC, the enzyme ‘E’ in Fig. 2a, left), but that cyclin degradation is distinct from MPF destruction. Goldbeter also posited a catalytic function for cyclin activation of MPF, without the positive feedback loop whereby MPF activates itself. Cyclin degradation in Goldbeter’s model is a two-step process, whereby MPF first activates the APC (‘E_a_’ in Fig. 2b, left), and then the APC drives cyclin degradation. In Tyson’s model, on the other hand, cyclin is a stoichiometric activator of Cdc2; i.e., cyclin binds with phosphorylated Cdc2 to form preMPF (Fig. 2c, left). preMPF is then converted into active MPF by the phosphatase activity of Cdc25. Because Cdc25 is activated by MPF, the conversion of preMPF to active MPF is a self-amplifying process in Tyson’s model. Tyson neglected the role of MPF in activating the APC; instead he assumed, without much experimental evidence, that only a phosphorylated form of cyclin was rapidly degraded.

Based on the reaction networks in Fig. 2 (left panels), the modelers converted their biochemical assumptions about the interactions of cyclin and MPF into differential equations for the rates of change of cyclin level and MPF activity. These mathematical equations describe the expected properties of the hypothetical mitotic control system given the assumptions behind each model, and these properties can then be compared with experimental observations to assess the reliability of the model. For instance, all three models exhibit spontaneous oscillations of MPF activity similar to MPF oscillations observed in frog egg extracts and to the mitotic cycles observed in early frog embryos. MPF oscillations in each model depend, of course, on the values assigned to the rate constants peculiar to each model, as illustrated in the middle panels of Fig. 2, which shows how the amplitude of MPF oscillations depends on the rate of synthesis of cyclin B (the rate constant *k*_s_). Notice that, in each model, the rate of cyclin synthesis must be greater than a certain minimum value for MPF oscillations to appear, as observed by Murray and Kirschner [6].

The models differ, however, in the dependence of MPF activity on a fixed concentration of cyclin (right panels of Fig. 2), which is Solomon’s signal-response curve (Fig. 1c). The Norel/Agur model is inconsistent with Solomon’s curve because the model has no ‘upper’ steady state of high MPF activity. In Goldbeter’s model, MPF activity increases abruptly over a small range of cyclin concentrations (a phenomenon called ‘ultrasensitivity’ [17]). Goldbeter’s ultrasensitive signal-response curve is consistent with the abrupt rise of MPF activity observed by Solomon. However, Goldbeter’s curve has a flat plateau for cyclin concentrations greater than the threshold, whereas Solomon’s measurements show a steady rise in MPF activity for increasing cyclin concentration above the threshold. In contrast to Goldbeter’s sigmoidal signal-response curve, Tyson’s model predicts an S-shaped curve, which is also consistent with Solomon’s experimental results, provided one interprets Solomon’s ‘threshold’ with the lower turning point of the S-shaped curve. Although both Goldbeter and Tyson referred to Solomon’s paper, neither of them commented on the significance of Solomon’s ‘cyclin threshold for Cdc2 activation’ with respect to their computed ‘signal-response’ curves.

The 1991 models were simple, with two or three differential equations (one for each time-varying species) and a handful of rate constants (one or two for each biochemical reaction). However, they were of little value in providing a unified, accurate picture of what was then known about mitotic regulation in eukaryotes and in making reliable predictions. To overcome these problems would require a more careful accounting of the molecular biology and cell physiology of frog eggs and yeast cells.

## Modeling starts from known causalities from which predictions are made

**Fig. 3:**
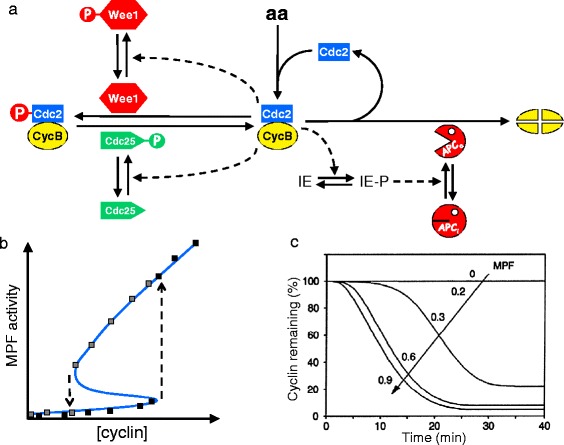
The Novak-Tyson 1993 model of mitotic control in frog eggs and extracts. **a** Molecular mechanism. Adapted from figure 1 of [18]. *Solid arrows* represent chemical reactions; *dashed arrows* represent catalytic activities. *IE* is an ‘intermediary enzyme’ phosphorylated by MPF. **b** Simulation of Solomon’s experiment in Fig. 1c. Adapted from figure 4B of [18]. For a fixed concentration of cyclin B, we plot the steady state concentration of MPF, according to the model. The *up arrows* and *down arrows* indicate irreversible transitions at the turning points of the S-shaped curve. For any fixed value of [cyclin] between the two arrows, the control system is ‘bistable’, i.e., it may persist in either of two alternative stable steady states (on the upper and lower branches) separated by an unstable steady state (the middle branch). The *black squares* recapitulate Solomon’s experiment [18]: starting from the steady state of low MPF activity, cyclin concentration is increased until the control system jumps to the steady state of high MPF activity. The *gray squares* recapitulate the experiments of Sha et al*.* [21] and Pomerening et al*.* [22]: starting from the steady state of high MPF activity, cyclin concentration is decreased until the control system jumps back to the low steady state. **c** Simulation of Félix’s experiment in Fig. 1d. This is figure 7a of [18]; used by permission. We simulated Félix’s experiment [13] by computing the rate of cyclin degradation when *k*_s_ = 0 (i.e., an ‘interphase-arrested extract’) and the initial concentration of MPF is increased from 0 to 0.9 (where 1.0 is the maximum possible MPF concentration in the model)

In 1993, we proposed a model of mitotic controls in frog eggs and extracts [18] that combined the best features of Tyson’s and Goldbeter’s models, namely, stoichiometric binding of Cdc2 and cyclin B, positive feedback loops through Cdc25 and Wee1, and delayed activation by MPF of the APC. Compared to the 1991 models, our 1993 model (Fig. 3) included more reactions among additional biochemical species (e.g., the regulatory enzymes Wee1 and Cdc25) and, hence, more differential equations to solve and more rate constants to estimate. In return, the added complexity allowed us to simulate in great detail many characteristic features of autonomous mitotic cycles in frog eggs [18] and of size-controlled division cycles in fission yeast [19, 20]. For example, the 1993 model accounts nicely for the classic experiments of Solomon et al. [12] and Félix et al. [13], as can be seen by comparing the simulations in Fig. 3b, c with the experimental results in Fig. 1c, d.

The successful features of the 1993 model gave us the confidence to make some intrepid predictions.

### First prediction (P1)

If MPF is indeed an S-shaped function of cyclin level, as suggested by Fig. 3b, then Solomon’s threshold reflects a discontinuous jump in MPF activity (the upward arrow in Fig. 3b), not a sigmoidal switch, as suggested by Goldbeter’s model (Fig. 2b, right panel). Consequently, if one were to start the extract in the upper steady state (the mitotic state) and decrease the cyclin concentration in stages, then one would observe a *lower* cyclin threshold for MPF *inactivation* (the discontinuous jump indicated by the downward arrow in Fig. 3b). According to our 1993 model (and Tyson’s 1991 model), there should be two different cyclin thresholds: one for flipping the switch ‘on’ at the up-arrow (Solomon’s threshold), and a lower threshold for flipping the switch ‘off’ at the down-arrow (a prediction of the model). For cyclin concentrations between the two thresholds, the switch can be either off (low MPF activity) or on (high MPF activity) depending on whether the switch started out in the off or on position. This sort of behavior — called ‘bistability’ — is familiar to anyone who has operated an old-fashion ‘snap-action’ light switch (as explained, for example, in Wikipedia), which toggles abruptly between lights on and lights off as the lever is pushed beyond the central position. When the lever is in the central position, the lights can be either on or off, depending on whether the lever is being pushed from the on position or from the off position. Our prediction that MPF activation is governed by a bistable switch differs radically from the simple interpretation of Solomon’s observations as a sigmoidal signal-response curve, for which MPF activation and inactivation occur at the same concentration of cyclin.

### Second prediction (P2)

Furthermore, if Solomon’s threshold corresponds to a discontinuous transition at the turning point of an S-shaped signal-response curve, then, if the cyclin concentration is only slightly larger than the threshold, the transition is still all-or-none but the time required to make the transition is very long (a phenomenon called ‘critical slowing down’ in the field of dynamical systems). Again, these properties are not characteristic of a sigmoidal switch.

### Third prediction (P3)

In addition, if MPF activation (at the interphase-to-mitosis transition) is governed by the turning point of an S-shaped curve, then the most natural explanation for checkpoint signals that delay this transition is a biochemical change that moves the turning point to larger values of total cyclin B.

### Fourth predicition (P4)

The most sensitive point in the mechanism for such a signal to act is the protein phosphatase that opposes MPF in the phosphorylation of Wee1 and Cdc25. This theoretical observation suggests a possible role for regulated serine/threonine protein phosphatases in cell cycle control.

At the time we made these predictions, there were no experimental observations to suggest that our predictions were correct and plenty of reasons to doubt them. Ten years later, predictions P1–P3 were confirmed experimentally by Sha et al. [21] and by Pomerening et al. [22]. Prediction P4 was not borne out by later molecular studies of checkpoint mechanisms, but regulated phosphatases, as we shall see, were found to play critical roles in other situations.

## The tension between parsimony and detail runs through systems biology like a fault line

Theoreticians, especially physicists, like simple, elegant models with few parameters (four parameters supposedly suffice to ‘fit an elephant’), but living matter, it would appear, is not so simple. The 1991 models of MPF regulation were parsimonious but inaccurate and ineffectual. Effective models of mitotic control (models that can account for a wide range of important physiological features and that can make trustworthy predictions) require dozen(s) of differential equations (one for each important protein and post-translationally modified form) and an attendant proliferation of unknown rate constants. Nonetheless, we were able to show [19] that the model in Fig. 3a, under quite reasonable conditions, can be reduced to just two differential equations and a few control parameters. This reduced model provides the desired accurate and parsimonious picture of many characteristic features of mitotic control in frog eggs and fission yeast cells. However, to dig deeper into the molecular foundations of cell cycle control, including regulation of DNA synthesis as well as mitosis, requires the creation of more detailed dynamical models, as illustrated by our later studies of fission yeast [23] and budding yeast [24].

## The principal disadvantage of a biologically detailed model is the parameter estimation problem

If we are compelled to build detailed models, then we must eventually face the problem of how to estimate dozens of unknown parameter values (rate constants, binding constants, enzyme activities, and so on). There is nothing new or unexpected about this problem; every rate constant is estimated ultimately by fitting a mechanistic model to experimental data. The more complicated the mechanism and the longer the list of unknown rate constants, the more data will be needed to estimate them. To estimate a large number of parameters, we will need reliable data that probe every aspect of the mechanism, but we do not need great quantities of data. A few data points on a time course of cyclin accumulation or disappearance may be enough to estimate the rate constant of cyclin synthesis or degradation with enough accuracy for our purposes. Indeed, we found, in fitting the 1993 model to frog egg-and-extract data, that we could estimate the important rate constants in the model with some confidence. One might consider these rate constant values as additional predictions of the model, since they were not, at the time, measured by independent biochemical investigations. Over the next few years, Kumagai, Dunphy and other biochemists, employing clever manipulations of frog egg extracts, provided direct measurements of many of the rate constants estimated by the model [25]. Although these biochemists were unaware of our model and certainly not intending to confirm our parameter values, their measured values are very much in line with our estimates [26].

## Mathematical models allow us to navigate with confidence far from the assumptions and reach surprising conclusions

The 1993 model led to a surprising conclusion, later confirmed experimentally, that entry into mitosis is an irreversible transition because it is implemented by a molecular mechanism exhibiting bistability (an S-shaped signal-response curve). The transition is triggered by cyclin concentration exceeding a threshold for MPF activation; after the transition is made, the cell remains in a mitotic state as cyclin concentration drops in anaphase and telophase, until the reverse transition (mitosis-to-interphase) is triggered at the other turning point of the S-shaped curve. In ensuing publications, we proposed that other cell cycle transitions (such as entry into S phase and exit from mitosis) are also controlled by bistable reaction networks [27], and these predictions have been confirmed in every case [28–31].

The model also predicts that spatial waves of MPF activation should propagate through multinucleate (syncytial) cells at a speed of ~50 μm per minute [19]. Although waves of mitosis traveling at the predicted speed are apparent in a 1974 film of nuclear divisions in an acellular slime mold [32], mitotic waves were only recently observed and accurately quantified by Chang and Ferrell [33] in frog egg extracts.

## The interesting question now is how various molecular components collectively give rise to phenotype and physiology

**Fig. 4:**
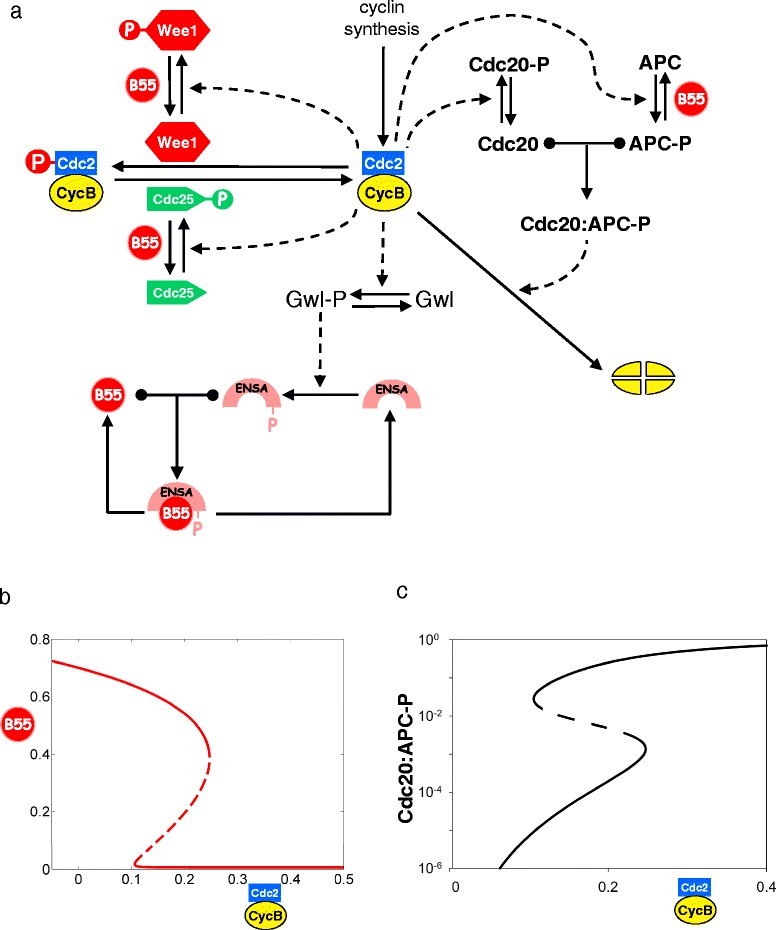
The regulation of protein phosphatase 2A during mitosis. **a** Molecular mechanism. Adapted from figure S1 of [38]. As in earlier figures, *solid arrows* represent chemical reactions; *dashed arrows* represent catalytic activities. The *T-shaped connector* with black dots on the crossbar and an arrowhead on the stem represents the reversible binding of two proteins to form a heterodimer. **b** Signal-response curve for PP2A:B55δ. Adapted from figure 5B of [38]. For a fixed activity of MPF we plot the corresponding steady state activity of PP2A:B55δ, the phosphatase that counteracts certain MPF-driven phosphorylations. The middle branch of the Z-shaped curve corresponds to unstable steady states. **c** Signal-response curve for APC. Figure S4 of [38]; used by permission. For a fixed activity of MPF we plot the corresponding steady state activity of APC, the ubiquitin-conjugating enzyme that labels cyclin B for degradation. Compare to panel (**b**)

Another surprising development is the recent recognition that one of the MPF-counteracting phosphatases, PP2A:B55δ, is regulated during entry into and exit from mitosis [34, 35]. This phosphatase (which we shall refer to as ‘B55’) is inhibited by the small proteins Arpp19 and endosulfine-α (which we will refer to collectively as Ensa). Ensa, in turn, is activated by phosphorylation by Greatwall kinase (Gwl), and Gwl is activated by phosphorylation by MPF (Cdc2:cyclin B) [36]. Experimental evidence suggested that B55 opposes the MPF-catalyzed phosphorylations of Wee1 and Cdc25 [34, 35] and of the APC [37], and we proposed (for theoretical reasons) that B55 is the Cdc2-counteracting phosphatase for Gwl as well. These known and predicted roles of B55 create a multitude of new positive and negative feedback loops in the network (Fig. 4a) with interesting dynamical consequences [38].

Of special significance is the double-negative feedback loop between Gwl and B55, which (we propose) creates a bistable switch controlled by MPF [38]. (Let’s call it the BEG switch, for B55-Ensa-Gwl [39].) As a cell enters mitosis, B55 activity is high, and high MPF activity is required to flip the BEG switch to the state of low B55 activity. Once the switch is flipped, moderate or even low activity of MPF is enough to keep B55 activity off and APC activity on (Fig. 4b). For this reason (we propose), as cells degrade cyclin B during anaphase and telophase, they do not prematurely inactivate the APC and abort cyclin B degradation. There is experimental evidence that APC activity functions as an ultrasensitive switch [40]; we propose that, on closer examination, APC will behave as a bistable switch (Fig. 4c) [38, 37].

If we correctly understand the role of regulated B55 in entry into and exit from mitosis, then one might reasonably ask how we could have achieved such a good description of mitotic controls in frog eggs with the 1993 model, which neglected any regulation of the MPF-counteracting phosphatase. The answer seems to lie in our choice of parameter values. In the 1993 model, we introduced a Cdc2-counteracting phosphatase (called ‘PPase’ in that paper, which we now know to be B55), and assigned it a moderate activity needed to set the limits of the bistable region (Fig. 3b) created by the interactions of MPF with Wee1 and Cdc25. Meanwhile, APC activation was controlled by a time-delay mechanism through the ‘intermediary enzyme’ IE in Fig. 3a. By adjusting the parameter values in the ‘IE-APC’ part of the network we could get APC to turn on after a time delay consistent with Félix et al. [13] (Fig. 3c) and to stay on long enough to degrade most of the cyclin B during the transition from metaphase to interphase. Compared to the 1993 model, the new model [38], with regulated B55, is parametrized differently and gives a better description (we believe) of the course of events at mitosis in frog embryos.

It is worth mentioning that the BEG switch is not essential for entry into mitosis in mammalian cells [39, 41]. In this sense, our 1993 model of frog egg extracts is actually more appropriate as a model of mammalian cells than of frog eggs, for which it was intended. Or we might say it is a model of frog eggs with constitutively phosphorylated Gwl.

The BEG mechanism was discovered because it is essential for mitotic entry in frog egg extracts [36, 42]. If Gwl and/or Ensa are depleted from extracts, then nuclei are blocked in late interphase (with replicated DNA). Why, we might ask, is Ensa essential in embryos but not in somatic cells? Recall that immature oocytes must arrest in G2 phase of meiosis I, awaiting a hormonal signal to undergo maturation. In this state, they may accumulate very high levels of preMPF, but they must not enter meiotic M phase prematurely. Having a high level of PP2A:B55δ could be a fail-safe mechanism for stabilizing the arrested oocyte. But in this case, B55 activity must be down-regulated in meiosis I in order for the egg to proceed into meiosis II, which might be the role of Gwl and Ensa.

## A mathematical model is a logical machine for converting assumptions into conclusions

In our experience, mathematical modeling has been an effective tool for investigating alternative hypotheses about the molecular mechanisms controlling the cell division cycle. The models showed how positive and negative feedback loops in the cyclin-MPF control system create toggle switches and oscillators that are crucial to the temporal sequencing of cell cycle events. Our 1993 model made many non-intuitive predictions about MPF activation in frog egg extracts, as described earlier. Later models of cell cycle controls in fission yeast [20, 23] and budding yeast [24] provided detailed accounts of the idiosyncratic phenotypes exhibited by mutant yeast strains, and suggested a new way to understand the irreversibility of cell cycle transitions in terms of bistable signal-response curves [27]. In most cases, the predictions of the models have been fully confirmed by subsequent experiments, as summarized in [43].

In general terms, building a model forces the modeler to lay out his or her assumptions clearly; analyzing and simulating the model determines the logical implications of these assumptions; and comparing the conclusions to experimental facts assesses the explanatory and predictive power of the mechanistic hypothesis. Where the conclusions of the model diverge from experimental facts may suggest problems with the ‘working hypotheses’ or missing components in the mechanism.

## Biology is complicated enough that we surely need every tool at our disposal

If mathematical modeling is such a nifty tool, why has it taken so long for molecular biologists to incorporate it into their armamentarium? In our experience, mathematical modeling of the cell cycle was at first politely ignored, then actively opposed, and lately grudgingly accepted. Perhaps this is to be expected of any new approach that is unusual and difficult to employ. But most people are comfortable now with the realization that molecular cell biology is a complex, interdisciplinary field that requires expertise from many quarters: genetics, biochemistry, nanotechnology, image processing, and bioinformatics, for example. Modern biological research is team-based, and, where appropriate, these teams need expertise in mathematical modeling of molecular regulatory networks. This is a specialized skill, much like X-ray crystallography, molecular genetics, proteomics or super-resolution microscopy. Life science departments need to acknowledge the vital role of models in biology and hire computational biologists who can bring this tool to bear on the important and difficult problems of today and tomorrow.
